# Editorial: Gender and Racial Bias in Sport Organizations

**DOI:** 10.3389/fsoc.2021.684066

**Published:** 2021-05-31

**Authors:** George B. Cunningham, Pamela Wicker, Nefertiti A. Walker

**Affiliations:** ^1^ Center for Sport Management Research and Education, Department of Health and Kinesiology, Texas A&M University, College Station, TX, United States; ^2^ Department of Sports Science, Bielefeld University, Bielefeld, Germany; ^3^ Mark H. McCormack Department of Sport Management, University of Massachusetts Amherst, Amherst, MA, United States

**Keywords:** diversity, equity, inclusion, gender, race, sport, intersectionality

## Overview

Legal mandates, social pressures for inclusion, and shifting demographic landscapes all contribute to an increased focus on diversity, equity, and inclusion in sport (Cunningham, 2019). Some leagues, such as the Women’s National Basketball Association, excel in this area, serving as a model for others (Lapchick, 2021). Despite the presence of exemplars, most of professional sport in the United States remains mired in the decades-long pattern of similarity and exclusion where White, able-bodied, cisgender, heterosexual men hold key leadership roles (Brassil & Lutz, 2020). These patterns are also evident in other sport contexts in the United States and around the world (Ahn & Cunningham, 2017; Cunningham et al., 2021; Walker & Bopp, 2011; Wicker et al., 2019; Wicker et al., 2020). Thus, even though members of underrepresented, minoritized groups frequently represent the majority of players, leadership roles are seemingly reserved for those who have historically held power.

In addition to limited access, members of underrepresented groups are likely to encounter stereotypes, prejudice, and treatment discrimination in sport. The disparities are evident among athletes, administrators, coaches, officials, and fans (Burton, 2015; Singer, 2016; Sveinson et al., 2019; Hindman & Walker, 2020; Wells et al., 2021; Wicker & Kerwin, 2020). These patterns suggest that, even though group diversity is frequently associated with desired outcomes, such as organizational effectiveness and positive affective outcomes (Lee & Cunningham, 2019), sport is a place where people who differ from the typical majority face various biases, limiting their access to and full participation in sport.

The purpose of this Research Topic was to explore these issues in greater depth. Specifically, we sought research from authors who 1) focused on taken-for-granted assumptions, 2) considered the myriad of factors that could influence the manifestation of bias, and 3) explored the intersections of race, gender, and other diversity forms. As we outline in the following section, the selected articles accomplished these aims.

## Selected Dimensions of Gender and Racial Bias in Sport Organizations

One of the themes to materialize from the articles was the value of critically examining the presence of and consequences of diverging from taken-for-granted assumptions and practices. Frick and Moser’s study offers an apt illustration, questioning the assumption that, among Nordic and Alpine skiers, women are less competitive than men. To do so, they analyzed decades of data from the sport. Their results showed that women and men were equally adept at managing career successes and failures, and that the career length of women and men was virtually identical. Thus, at least among elite skiers, their findings counter the notion of gender differences in competitiveness and drive. From another perspective, Braumüller et al. drew from a large-scale dataset, which included respondents from Germany, Scotland, Austria, Italy, and Hungary, to explore the experiences of transgender, non-binary, and cisgender athletes. Given that sport is largely segregated based on sex assigned at birth, and transgender and non-binary athletes challenge this demarcation, it is possible they have poor experiences in sport. Consistent with this perspective, results showed that transgender and non-binary athletes faced continued anti-trans bias, including structural forms of discrimination.

Two articles considered factors that might influence the presence of bias in sport. Mire et al., for example, conducted a study of weightlifters and examined whether coach-athlete gender similarity influenced the athletes’ performance. Among men, gender congruence was associated with better performance. Women performed better when their coach was a man, but only until age 43, at which point they performed better when guided by a woman. The authors noted historical biases against women in the sport could contribute to these patterns. Demographic similarity, or a lack thereof, is also associated with referee decisions. Specifically, in examining multiple years of data from the National Football League, Eiserloh et al. found that Black umpires called more penalties when their referee (the leader of the team of officials) was White. The authors reasoned that Black umpires might feel more pressure to assess infractions when their team leader is White—stresses others have observed in different sport contexts (Foreman & Turick, 2020).

The importance of contextual factors was highlighted in two studies. Focusing on men’s intercollegiate basketball in the United States, Nesseler et al. found that Black coaches were underrepresented—a pattern that continued for decades. The effects were more pronounced, however, in Division III institutions, which are comparatively smaller with more White undergraduate students. Gomez-Gonzalez et al. also illustrated the importance of context. The authors noted that previous researchers had found that demographic dissimilarity was associated with the number of infractions a referee called on an athlete. Most of these studies, though, were set in the United States or United Kingdom and focused on men’s sport. Gomez-Gonzalez et al. diverged from this pattern, analyzing data from women’s basketball teams in Spain. Contrary to previous work, the authors observed no effects of racial dissimilarity or nationality dissimilarity. Thus, the country and sport might moderate the relationship between dissimilarity and infractions called.

Finally, other contributors highlighted the importance of explicitly considering intersectionality—a position for which previous researchers have advocated (Walker & Melton, 2015). In the first study, Bartsch and Rulofs focused on physical education teachers’ attitudes toward children from refugee backgrounds. Racialized and gendered notions of threat and vulnerability were evident, with four themes emerging: *victimization and vulnerabilization, notions of threat and impulsivity, claims for assimilation and normalization*, and *demands for discipline*. Given the increased number of refugees around the world, and the important role of physical activity and sport in their lives (Anderson et al., 2019), Bartsch and Rulofs’ findings are especially instructive. Finally, Cooper and colleagues made a persuasive argument for new, innovative leadership approaches in sport. They argued that leaders should adopt anti-racism, anti-sexism, and culturally responsive stances. Only through such a paradigmatic shift can leaders hope to create and maintain diversity, equity, and inclusion in their sport organizations.

## Synthesis

The Research Topic enhances the understanding of gender and racial bias in sport and its organizational settings. The included articles have provided rich insights into the topic from a number of perspectives, including the challenge of taken-for-granted assumptions, the study of factors that might influence the presence of bias in sport, the role of contextual factors in terms of national research settings, and the consideration of intersectionality. They help understanding how, why, and in what contexts gender and racial bias toward and discrimination of under-represented groups in sport is evident, ultimately enhancing the evidence base for taking informed action toward making sport a more inclusive and diverse environment.
